# New York Letter

**Published:** 1896-08

**Authors:** Ogden C. Ludlow

**Affiliations:** 2309 Seventh Avenue; New York


### NEW YORK LETTER.

NEW YORK, July 15, 1896.

In glancing back over the recent work of our medical societies, one is impressed with the great activity of those devoted to specialties as compared with those not so limited. It would seem as if one of the familiar axioms found in our books on mathematics had, after all, become twisted in some way, and that a part must now be considered greater than the whole. We are reminded of an amusing "card," now going the rounds of the medical press, in which a physician advertises that he is engaged in a new specialty, "general medicine." There is a deal of truth and food for sober thought in this joke, for, in this city at least, specialism runs riot. It may be permissible, however, to refer to a topic of some general interest in this "new specialty"—I refer to the treatment of heart disease.

This has recently been made the subject of papers by Dr. W. H. McEnroe and Dr. William H. Thomson. The former called attention to the benefit to be derived from combination of well known cardiac stimulants. Thus, digitalis, by prolonging the systole and shortening the diastole, causes the heart to remain small while under its influence, but its effect in causing a contraction of the arterioles, and so increasing the peripheral resistance, must be neutralized. This is accomplished most effectually by combining nitroglycerine with the digitalis, in doses not exceeding $\frac{1}{100}$th of a grain. It is perhaps not very widely known that iodide of potassium, in doses of five grains three times a day, freely diluted with milk, is an excellent vascular stimulant, although this action is rather slowly developed. Where it is indicated, nitrate of amyl may be given freely. Dr. McEnroe said that he had known as much as sixty minims to have been given at one time, by inhalation, without any harmful result, and with positive benefit to the patient. For the insomnia of advanced heart disease, morphia is the best remedy. It should be given in doses of one-fourth of a grain, combined with twenty grains of the bromide of sodium. Where constipation or other conditions make it desirable to avoid morphine, one-fourth to one-half grain of codeine may be substituted. Trional will be found an excellent hypnotic, in doses of ten grains, alone or combined with a small dose of morphine. Dr. Thomson said that for a right understanding of the treatment of heart disease, we should divide these cases into primary and secondary. Primary heart disease arises almost exclusively from rheumatism and chorea. The younger the patient, the more likely is a slight cardiac lesion to develop into a serious structural alteration. The reason that the endocardium is so much more liable to become the seat of fibrinous deposit and permanent lesion than are the joints, as a result of acute rheumatism, is that the heart is in incessant motion. The first slight fibrinous deposit becomes a source of constant irritation, just as do the granulations on the eyelid, and, as a result, the lesion of the endocardium is progressive. In the treatment of primary heart disease, the first place should be given to rest in bed.

Where this alone is not sufficient to bring down the pulse-rate, and so secure to the heart the much-needed rest, aconite should be administered. It is not only a cardiac sedative, but is capable of markedly relieving inflammatory pain. It will be found most valuable in slowing the heart's action and relieving the dyspnea, and, like other vegetable nervines, its action on the heart is no more permanent than that of tobacco. For the dyspnea of mitral stenosis belladonna will be found useful, as the characteristic action of this drug is to restore rhythmical action to unstriped muscular fiber. It is advantageously combined with strophanthus and sparteine. The prophylactic treatment of cardiac disease should not be overlooked. Cases of acute rheumatism and of chorea should be treated by prolonged rest in bed, for the cardiac lesion is often slow in announcing its presence. This is particularly true of chorea, and hence it is of great importance that the prudent physician should carefully examine the heart of such patients at intervals after apparent recovery from chorea. It will be found sometimes that the patient will recover from the chorea with no evidence of disease of the heart, and yet, after an interval of perhaps one or two years, there will be evidence that the heart has been damaged. It is hardly necessary, therefore, for the writer to emphasize the advice given regarding the keeping of such patients under medical supervision for a considerable time—"forewarned is forearmed." Again, rheumatic individuals should be taught the importance of always protecting the body from chilling by wearing good flannels, both by night and day. This seems to be a trite remark, but we think it would not be difficult for most of us to recall instances in which patients have been very careful about wearing flannel as a protection to the skin in the daytime, but who have discarded such protection at night, wholly unmindful of the many opportunities for catching cold during sleep by tossing off the clothes and exposing the surface of the body to draughts of cool air. Dr. Thomson reminds us that when a rheumatic individual develops the first sign of a tonsillitis, much can be accomplished in the way of prophylaxis by the application of tincture of iodine to the throat, and the prompt internal administration of the salicylates. In all cases of chronic heart disease, he says, we should bear in mind the evil effect of anemia; but this does not necessarily imply that we must pour iron into these patients, simply because we have discovered that their mucous membranes are pallid. In febrile anemias, such as phthisis, rheumatism, and pernicious anemia, iron is likely to do more harm than good. The blood corpuscles may be fed in other ways. In rheumatic anemia especially, cod-liver oil is more apt to prove of benefit than iron, and occasionally arsenic will lend a helping hand. Dr. Thomson emphasizes the fact that the secondary diseases of the heart generally begin in disturbances of the peripheral circulation. As a result the heart undergoes hypertrophy, and subsequently degeneration and dilatation. In the therapeutics of this class, fresh air must be given a prominent place. Hours spent in a dry, equable air give strength to the weakened cardiac muscle; and this should be the object of our treatment. When the heart becomes dilated, the pulse grows rapid, not as a result of irritation of the heart itself, but of the inability of the heart to empty itself at each stroke. Here, instead of prescribing rest in bed and aconite, we should advise systematic and gradually increasing exercise, and internally digitalis. After a few weeks of properly applied massage, with proper resistance on the part of the patient to these movements, Dr. Thomson says he has seen a very marked improvement in the breathing. The digitalis should be combined with nitroglycerin, and its action is often greatly assisted by giving about half a grain of calomel, three times a day, up to signs of mercurialization. Opium is a most valuable heart stimulant for occasional use. These cases are often kept on a fluid diet, but it should be borne in mind that the cardiac dropsy may be made to disappear by restricting the quantity of fluid ingested.

Another subject, always interesting and of practical bearing to the general practitioner, is obstetrics. Dr. J. Milton Mabott, in a recent brief paper, stated what he styled "Three Warnings of Interest to Obstetricians." The first was, that physicians should always warn pregnant women under their care to report at once the occurrence of any hemorrhage from the vagina, however slight. He had seen two deaths due to a neglect of this precaution. He next advises the physician to warn parturient women to refrain from handling their genitals during parturition and the puerperium, as he believes that he has met with a fatal case of pyemia due to such self-examination. His last plea was that it was the duty of the physician to warn nursing women that they should never allow themselves to fall asleep with the baby at the breast. His reason for uttering this warning was, that deaths from overlying were almost criminally common. Providing the infant with a separate bed had been found to not entirely remove this danger. In the discussion of this paper, Dr. E. A. Tucker said that the good sense of these warnings could not be disputed, but as he had known an intelligent nurse to nearly lose her life from a failure to report the occurrence of hemorrhage during pregnancy, he was skeptical as to the good results to be attained by pointing out these dangers. Dr. P. A. Harris called attention to the rarity of cases of puerperal septicemia among Southern women attended by the negro midwives, among whom there was an unwritten law forbidding vaginal examinations. Dr. Brooks H. Wells said that the same thing had been noticed among the Indians of the West; the refraining from making vaginal examinations apparently more than counterbalanced the danger of being confined in their filthy hovels. Dr. Marx said that he thought some cases of puerperal sepsis, occurring among the lower classes, arose from indulgence in sexual intercourse shortly after labor.

Under the title of "*Non nocere* in Obstetrics," Dr. Julius Rosenberg spoke of certain meddlesome practices in modern midwifery which had received the sanction of high authority. In the relentless war now waged against pathogenic germs it is not uncommon for the combatants to go beyond the bounds of prudence and common sense, and certain thoughtful obstetricians of the present day believe that this remark applies with perhaps greater force to obstetrics than to general surgery. Thus, as Dr. Rosenberg says, it has been found that in normal pregnancies the vaginal secretion is not only free from pathogenic germs, but is inimical to germ life, yet many obstetricians insist upon giving an antiseptic vaginal douche at the beginning of labor. To this he objects, as well as to the routine use of such douches after labor, in the absence of distinct indications for them. He says, and I think the experience of many others will indorse the statement, that these douches are often the very cause of the woman becoming infected. Another example of modern meddlesome midwifery, Dr. Rosenberg thought, was to be found in the practice of subjecting to operation cases in which pregnancy is complicated by uterine fibroids. He had attended six such cases, and has saved the lives of all the mothers, and of all but one child. Statistics of operative interference, he said, could not make as good a showing. Although these fibroids are often quite large during pregnancy, they are prone to diminish after parturition. That his personal experience should not stand alone as an argument for non-interference in these cases, he had searched the literature of the subject, and had, he thought, found sufficient justification for the statement, that fibroid tumors do not seriously complicate many cases of pregnancy.

In discussing the paper, Dr. Robert A. Murray said that there were many germs in the vagina, and if these were not removed by an ante-partum douche of some antiseptic solution, infection was liable to occur through a laceration of the canal. Unless the external genitals were carefully cleansed, the examining finger could not long remain aseptic.

Dr. Rosenberg replied that in tenement-house practice, he believed that antisepsis in obstetrics should be restricted to the examiner's hands and the woman's genitals.

To these statements the writer would add that in his humble opinion much more emphasis should be laid upon the dangers of post-partum curetting, in this class of practice particularly. Without wishing in any way to belittle the beneficent results obtained by such curetting, he thinks that more consideration should be given to its dangers. There are not lacking obstetricians of vast experience and excellent judgment, who, though thoroughly alive to the great advances made in modern times in the obstetric art, feel very strongly on this point. They have seen a post-partum curetting, for the removal of a small piece of placenta, followed by a chill, high fever, and all the evidences of fresh and more extensive infection, and they have observed these symptoms and signs aggravated by another curettement. It has been all too plain that the curetting has served chiefly to open new avenues to infection. It may be urged that this should not occur. Granting this, and also that it rarely does happen *under favorable surroundings and conditions*, still it cannot be denied that it does occur much too commonly in obstetric practice among the poor, and that, too, when the curettement has been done by well-known and experienced operators. The writer has recently heard of a fatal case under just such circumstances, and it is by no means unique.

2309 *Seventh Avenue.* Ogden C. Ludlow, M.D.

---

### Fistula in Ano.

Operate upon all cases where there is sufficient vitality to heal the wound. Always divide the fibrous membrane at the bottom of the tract, and pack the wound for the purpose of healing with granulation. Always open abscesses early to prevent fistula in ano; when operating in a tuberculous patient, give him the benefit of the doubt. Never fail to examine thoroughly for small branches leading out from the main tract, and examine for an associated stricture which may be the cause of the fistulous tract. Never cut the sphincter but once in any operation, and beware and warn the patient of the danger of incontinence. Confine the patient in bed, and do not trust his care exclusively to a nurse; tubercular cases are an exception in that they demand moderate exercise and fresh air. Physiological rest is the first principle for the relief of all diseases. In all operations involving the rectum, it is good surgery to dilate the sphincter. All cases of fistula in ano should be operated upon preferably by the knife, except where Bright's disease, cancer, cardiac or hepatic disease exists.—B. B. Porter, in *American Journal of the Medical Sciences.*

---

In cases of severe injury to fingers by laceration or contusion, put the entire hand into a very ample soaking-wet dressing. Do not even trim off a piece of flapping skin. Incision for drainage is all that is allowable until healing is very well under way or even quite complete. You may then look over the ground and see whether it is worth while to sacrifice anything. A half-inch of *boneless* finger may be of incalculable value to its possessor.—*International Journal of Surgery.*

